# Thanks to Reviewers 2016

**DOI:** 10.5334/pb.376

**Published:** 2016-12-28

**Authors:** Editorial Team

**Affiliations:** 1 Psychologica Belgica, Belgium

## Abstract

All manuscripts published in *Psychologica Belgica* have been assessed conscientiously and unselfishly by expert reviewers. The quality of our journal totally depends on their valuable and constructive criticisms to the authors. Both the editors and the authors highly appreciate the input and dedication of all our reviewers. Many thanks.

**Katarzyna Adamczyk**, Adam Mickiewicz University in Poznań, Poland

**Nathalie Aelterman**, Ghent University, Belgium

**Stefan Agrigoroaei**, Katholieke Universiteit Leuven, Belgium

**Alejandra Alarcon**, Université Libre de Bruxelles, Belgium

**Cristina Antunes**, University of Trás-os-Montes and Alto Douro, Portugal

**Matthijs Bal**, University of Bath, United Kingdom

**Moti Benita**, Ben Gurion University of the Negev, Israel

**Wim Beyers**, Ghent University, Belgium

**Boris Bizumic**, Australian National University, Australia

**Liesbet Boone**, Ghent University, Belgium

**Maarten Boudry**, Ghent University, Belgium

**Dries Bostyn**, Ghent University, Belgium

**Piet Bracke**, Ghent University, Belgium

**Lieven Brebels**, Katholieke Universiteit Leuven, Belgium

**Asteria Brylka**, University of Northampton, United Kingdom

**Beiwen Chen**, Ghent University, Belgium

**Oliver Christ**, Fern Universität in Hagen, Germany

**Elien De Caluwé**, Ghent University, Belgium

**Kathleen De Cuyper**, Katholieke Universiteit Leuven, Belgium

**Stephanie De Oliveira Laux**, University of Osnabrück, Germany

**Egon Dejonckheere**, Katholieke Universiteit Leuven, Belgium

**Ellen Delvaux**, Katholieke Universiteit Leuven, Belgium

**Kristof Dhont**, University of Kent, United Kingdom

**Andrew Elliot**, University of Rochester, USA

**Chiedu Eseadi**, University of Nigeria Nsukka, Nigeria

**Ernestina Etchemendy**, Universidad de Valencia, Spain

**Sarah Galdiolo**, Katholieke Universiteit Leuven, Belgium

**Albert Garcia-Romeu**, Johns Hopkins School of Medicine, USA

**Patrick Gaudreau**, University of Ottawa, Canada

**Nicolas Gillet**, Universite Francois-Rabelais de Tours, France

**Leen Haerens**, Ghent University, Belgium

**Alexandre Heeren**, Katholieke Universiteit Leuven, Belgium

**Céline Hinnekens**, Ghent University, Belgium

**Marlies Houben**, Katholieke Universiteit Leuven, Belgium

**Konrad Jankowski**, University of Warsaw, Poland

**Olivier Klein**, Université Libre de Bruxelles, Belgium

**Theo Klimstra**, Tilburg University, Netherlands

**Fleur Kraanen**, de Waag, Netherlands

**Fransciska Krings**, Université de Lausanne, Switzerland

**Giovanna Leone**, Università di Roma, Italy

**Craig Leth-Steensen**, Carleton University, USA

**Christophe Leys**, Université Libre de Bruxelles, Belgium

**Lisa Linnenbrink-Garcia**, University of Michigan, USA

**Olivier Luminet**, Katholieke Universiteit Leuven, Belgium

**Koen Luwel**, Katholieke Universiteit Leuven, Belgium

**Koen Luyckx**, Katholieke Universiteit Leuven, Belgium

**Geneviève Mageau**, University of Montreal, Canada

**Lars-Erik Malmberg**, University of Oxford, United Kingdom

**Henri Markovits**, Université du Québec à Montréal, Canada

**Andrew Martins**, The University of Sydney, Australia

**Dora Matzke**, University of Amsterdam, Netherlands

**Cecil Meeusen**, Katholieke Universiteit Leuven, Belgium

**Gaetan Mertens**, Ghent University, Belgium

**Aikaterini-Aliki Michou**, Bilkent University, Turkey

**Anneleen Mortier**, Ghent University, Belgium

**Oana Negru**, Babeş-Bolyai University, Romania

**Greta Noordenbos**, Universiteit Leiden, Netherlands

**Soren Ostergaard**, Aarhus University Hospital, Denmark

**Thea Peetsma**, University of Amsterdam, Netherlands

**Paraskevas Petrou**, Erasmus University Rotterdam, Netherlands

**Maria Ranzini**, Université libre de Bruxelles, Belgium

**Catherine Ratelle**, Université Laval, Canada

**Guy Roth**, Ben-Gurion University of the Negev, Israel

**Rachel Seginer**, University of Haifa, Israel

**Corwin Senko**, University of New York, USA

**Dirk Smits**, Odisee University College, Belgium

**Marie Stievenart**, Université de Liège, Belgium

**Charles Stoned**, John Jay College, USA

**Catia Teixeira**, Katholieke Universiteit Leuven, Belgium

**Kiran Vanbinst**, Katholieke Universiteit Leuven, Belgium

**Jasper Van Assche**, Ghent University, Belgium

**Hans van der Baan**, University of Amsterdam, Netherlands

**Anja van der Voort**, Universiteit Leiden, Netherlands

**Hilde Van Keer**, Ghent University, Belgium

**Steven Verheyen**, Katholieke Universiteit Leuven, Belgium

**Jan Verplaetse**, Ghent University, Belgium

**Ralf Wolfer**, University of Oxford, United Kingdom

**Sofie Wouters**, Katholieke Universiteit Leuven, Belgium

**Vincent Yzerbyt**, Katholieke Universiteit Leuven, Belgium

**Mingming Zhou**, University of Macau, China

